# Adaptability of the load sharing between the longissimus and components of the multifidus muscle during isometric trunk extension in healthy individuals

**DOI:** 10.1007/s00421-023-05193-5

**Published:** 2023-04-20

**Authors:** Louise Tier, Sauro E. Salomoni, François Hug, Manuela Besomi, Paul W. Hodges

**Affiliations:** 1 School of Health and Rehabilitation Sciences, The University of Queensland, Brisbane, Qld 4072 Australia; 2 LAMHESS, Université Côte d’azur, Nice, France; 3 Institut Universitaire de France (IUF), Paris, France; 4 School of Biomedical Sciences, The University of Queensland, Brisbane, Australia

**Keywords:** Electromyography, Shear wave elastography, Coordination, Back extension

## Abstract

**Purpose:**

Redundancy of the musculoskeletal system implies multiple strategies are theoretically available to coordinate back extensor muscles. This study investigated whether coordination between back muscles during a tightly constrained isometric trunk extension task varies within and between individuals, and whether this changes following brief exposure to activation feedback of a muscle.

**Methods:**

Nine healthy participants performed three blocks of two repetitions of ramped isometric trunk extension in side-lying against resistance from 0–30% of maximum voluntary contraction over 30 s (force feedback). Between blocks, participants repeated contractions with visual feedback of electromyography (EMG) from either superficial (SM) or deep multifidus (DM), in two conditions; ‘After SM’ and ‘After DM’. Intramuscular EMG was recorded from SM, DM, and longissimus (LG) simultaneously with shear wave elastography (SWE) from SM or DM.

**Results:**

In the ‘Natural’ condition (force feedback only), group data showed incremental increases in EMG with force, with minor changes in distribution of activation between muscles as force increased. SM was the most active muscle during the ‘Natural’ condition, but with DM most active in some participants. Individual data showed that coordination between muscles differed substantially between repetitions and individuals. Brief exposure to EMG feedback altered coordination. SWE showed individual variation, but findings differed from EMG.

**Conclusion:**

This study revealed substantial variation in coordination between back extensor muscles within and between participants, and after exposure to feedback, in a tightly constrained task. Shear modulus revealed similar variation, but with an inconsistent relationship to EMG. These data highlight highly flexible control of back muscles.

## Introduction

Muscle coordination reflects the distribution of force amongst individual muscles to produce a motor task (Hug and Tucker 2017). As the trunk involves a complex array of muscles with synergist actions, different muscle coordination strategies can be adopted to achieve a motor goal (Claus et al. 2018), referred to as redundancy (Bernstein 1966). Previous studies suggest differences in back muscle coordination between individuals (Eriksson Crommert et al. 2017), and the potential for changes in coordination within an individual in response to specific stimuli (e.g. nociceptive stimuli (Hodges et al. 2013)). Whether variation in back muscle coordination is explained by subtle differences in the task that is performed (i.e. different goal), or differences in muscle coordination for an identical task, could be answered by evaluation of a tightly constrained isometric trunk extension task.

The paraspinal muscles are synergist trunk extensors, but anatomy underpins variation in their potential to generate extension torque and other actions. For instance, the deep muscle fibres of multifidus (DM) crosses few segments and is close to the centre of rotation of vertebral segments, offering limited potential to generate extension torque, and instead playing a more significant role in intervertebral control (Hodges and Danneels 2019; Schuenke et al. 2015). The superficial fibres of multifidus (SM) arise from the spinous process, descend over multiple segments and control lumbar extension (Hodges and Danneels 2019; Schuenke et al. 2015). The longer, more lateral erector spinae (ES) muscles cross many segments, including fibres that extend the entire thoracic and lumbar spines (Eriksson Crommert et al. 2017; Macintosh and Bogduk 1987; Schuenke et al. 2015). As predicted from anatomy, activation levels differ between these muscles in many tasks (Claus et al. 2018; MacDonald et al. 2006; Moseley et al. 2002). The coordination between muscles depends on how trunk extension is performed. For instance, when asked to sit upright, participants show different muscle activation patterns that depend on the between-individuals differences in pelvic alignment and spinal curvatures (Claus et al. 2018). It is plausible that constraint of a trunk extension task to favour the mechanical action of one muscle would limit the variation between individuals and between repetitions within an individual, but this requires investigations.

Task constraint does not always induce a consistent use of the most energy efficient muscle coordination strategy. For instance, studies of forearm muscles have reported that when task constraints were changed, habitual coordination strategies persisted regardless of their lower efficiency (de Rugy et al. 2012). Back muscle coordination might also not be predictable based on mechanical efficiency leading to high variation between individuals (based on individual experience), but low within-individual variation.

Other work highlights the potential benefits of variation in motor strategy *within* individuals for reasons such as exploration of movement options (Dingwell et al. 2001), variation of loading (Hodges and Tucker 2011) and injury minimisation (Hamill et al. 1999). One way to probe the potential for variation in coordination of back muscles in a constrained task would be to test the response to subtle stimuli, such as brief exposure to feedback of an individual muscle’s activation.

A comprehensive understanding of coordination between muscles requires measurement of activity and mechanics of individual muscles. Electromyography (EMG) provides insight into activation, but cannot measure mechanical properties. Shear wave elastography (SWE) can estimate tissue stress and has been applied to back muscles (Murillo et al. 2019; Tier et al. 2021). Although both EMG and SWE have limitations, together they could provide insight into coordination between back muscles.

This study aimed to investigate coordination (which we define as the relationship between amplitude of activation of muscles) amongst three back muscles (SM, DM and longissimus (LG)) during a constrained isometric trunk extension task. The specific aims were to: (i) evaluate each individual’s ‘Natural’ strategy for muscle coordination in this constrained task, (ii) quantify variation in the ‘Natural’ strategy within and between participants, (iii) evaluate whether coordination is modified after brief EMG feedback of one muscle, (iv) evaluate whether variability within and between participants differs after exposure to feedback and (v) compare SWE and EMG measures made for SM and DM.

## Methods

### Participants

Eleven healthy individuals participated (two were excluded because of inaccurate data synchronisation or BMI > 26), (five men; age 30 ± 5 years; height 170 ± 11 cm; weight 68 ± 12 kg; BMI 23 ± 2 kg/m^2^). Exclusion criteria were: history of LBP which limited function or required treatment, spinal or lower limb surgery, bleeding or clotting disorders or pregnancy. The Institutional Medical Research Ethics Committee approved the study and participants provided written informed consent. Participants were involved in another study (Tier et al. 2021).

### EMG

EMG was recorded using bipolar intramuscular fine-wire electrodes fabricated from Teflon-coated stainless-steel wire (two strands [75 mm diameter] with 1 mm of insulation removed, bent to form hooks at 2 mm and 3 mm) and threaded into hypodermic needles (DM-70 mm×23G; SM-50 mm×23G; LG-32 mm×23G). Electrodes were inserted under ultrasound guidance into the right SM and DM adjacent to L4/5, and LG at L2 (Claus et al. 2018). EMG was amplified 1000 times, band-pass filtered between 10–1000 Hz (Neurolog, UK) and sampled at 2000 Hz using a Power 1401 data acquisition system with Spike2 software (Cambridge Electronic Design, UK).

### SWE

**Fig. 1:**
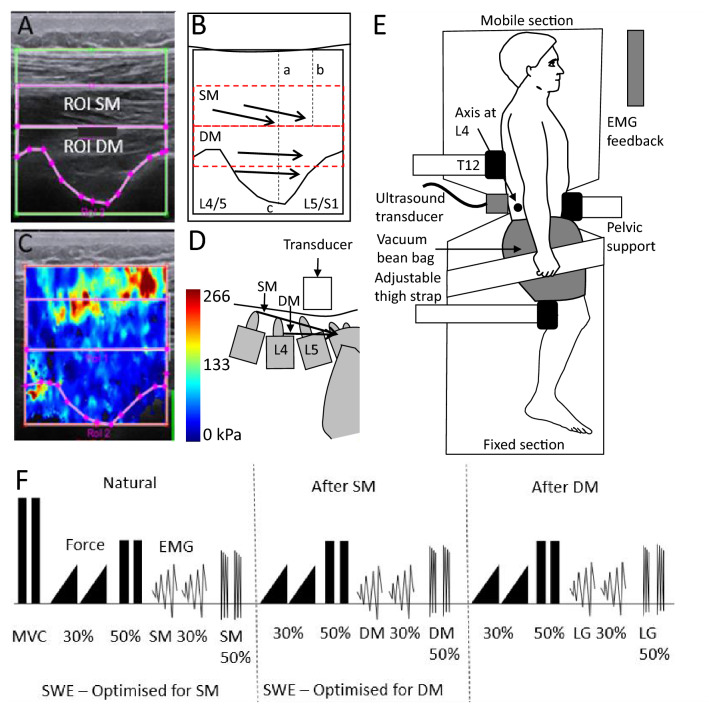
Methods. **A** B-mode ultrasound image with regions of interest (ROI) identified at rest for superficial (SM) and deep fibres (DM) of the multifidus muscle. The image is shown for the ‘After SM’ condition when the image was optimised for DM. **B** Line drawing of B-mode ultrasound image of anatomical landmarks of L4/5 and L5/S1 facet joints, used to define the two distinct ROIs of the multifidus (a—resting thickness of SM, b—50% SM thickness, c—deepest point between adjacent facet joints). The black arrows depict muscle fibre direction. The red dotted lines depict the area of DM (bottom) that was available for analysis in the ‘Natural’ condition when imaging was optimised to SM (top). **C** Elastogram during contraction at 20% maximum voluntary contraction (MVC). **D** Illustration of orientation of the fascicles of DM and SM relative to the transducer. **E** Experimental setup for the side-lying isometric trunk extension task. Axis of the pivot table was aligned approximately to the L4-5 joint. **F** Sequence of tasks indicating periods with force (black triangles) and EMG (zig-zag lines). Periods with shear wave elastography optimised to the DM or SM are identified. Participants completed two repetitions of MVC, followed by two 30% MVC ramped force-feedback contractions over 30 s and two 50%MVC hold contractions. EMG feedback trials included two ramp and two hold contractions (data not presented here)

Shear modulus was estimated using an ultrasound device (Aixplorer, SuperSonic Imagine, France) coupled with a linear transducer (SL10-2; SuperSonic Imagine, France). Elastography maps were recorded at 0.7–1.3 Hz with a spatial resolution of 1 × 1 mm. Recordings were made of SM and DM (not LG) with the transducer location and elastography maps optimised to image the multifidus muscle as previously described (Tier et al. 2021). Briefly, the transducer was positioned ~ 20 mm lateral to L4 spinous process with the caudal edge rotated ~ 10–20° laterally to align with muscle fibres at rest (different for each muscle). The elastography map included muscle between the superficial fascia and region between L4/5 and L5/S1 facet joints throughout contraction (Fig. 1). In the ‘Natural’ condition: the transducer was optimised for SM (which included DM) (elastography map: 35–38 mm wide × 30–32.5 mm deep). For the ‘After SM’ condition: the image was optimised for DM, but also included SM in a slightly different location to the ‘Natural’ condition (elastography map: 30 mm wide × 28-29 mm deep).

### Procedure

Participants were positioned in side-lying on a pivot table (axis at L4), with hips and knees flexed to 30°. Padding and restraints were used to reduce but not eliminate potential contribution from the lower limbs and to accommodate individual variation in soft tissue. The position of the pelvis was held rigidly with padded supports in front of the pelvis (across anterior superior iliac spines) and behind the knees, and a vacuum bean bag and strap that surrounded the proximal thigh to restrict potential for leg force to contribute to the task. A rigid padded bar at T12 was fixed to the mobile (upper) section of the table, which was connected by a rigid cable and strain gauge (GS-KMT Load Cell, Gedge Systems, Australia) to a stable frame (Fig. 1E).

The starting position was the naturally adopted trunk angle, (e.g. trunk muscles relaxed) without tension in the force cable. Participants isometrically extended the trunk from lumbopelvic junction by exerting force into the bar at the bony prominence of T12 across the width of the back. Participants performed two 4-s maximum voluntary isometric contractions (MVC) of back extension, separated by a 2-min rest. EMG data were presented as root mean square (RMS) amplitude (500 ms time constant) and its highest value along with highest force was recorded. After baseline subtraction, 30% values were calculated for used as targets for ramped contractions. Maximal EMG values were used for EMG normalisation (see below).

Participants performed two repetitions of ramped isometric extensions from rest to 30% of MVC force (force feedback displayed on a monitor) in three conditions (Fig. 1F), with verbal instruction provided to match the target but no additional detail of how to complete the task:

1. ‘Natural’: initial two repetitions of the task without exposure to any muscle feedback.
2. ‘After SM’: immediately after two repetitions of trunk extension using EMG feedback from SM from 0–30% EMG MVC over 30 s and two contractions held at 50% EMG MVC for 6 s.
3. ‘After DM’: immediately after two repetitions of trunk extension using EMG feedback from DM from 0–30% EMG MVC over 30 s and two contractions held at 50% EMG MVC for 6 s.

All participants performed the task in the same order except for one who performed ‘After DM’ before ‘After SM’ (no shear modulus data was available for ‘After SM’). Shear modulus was recorded from SM/DM during ‘Natural’ and ‘After SM’ conditions and synchronised with EMG using a trigger that activated when imaging commenced.

### Data analysis

EMG data were analysed using MATLAB (MathWorks, Inc., Natick, MA). EMG was band-pass filtered between 50–1000 Hz (dual-pass, zero-lag 4th order Butterworth filter) and assessed from 0.5 s before force ramp onset until completion. RMS EMG amplitude was calculated for each 1% MVC increment in force (between 0.5% before and 0.5% after each increment). Brief fluctuations in force (< 200 ms) and segments with movement artefact were excluded. RMS EMG was averaged for Bins of 5% force increments (Bin1 1–5%, Bin2 6–10%, …Bin6 26–30%). EMG amplitude was normalised to the highest RMS EMG obtained during MVC (250 ms). Change in EMG was calculated as the difference between consecutive Force Bins (ΔBin1: (Bin1-baseline) …ΔBin6: Bin6−Bin5). Average EMG for each muscle was expressed as a percentage of total normalised EMG across all muscles (%Total).

For analysis of individual variation between repetitions in the ‘Natural’ condition, for each participant, the muscle with the highest normalised EMG amplitude and percentage variation (difference of average EMG between repetitions for each muscle) was calculated. To investigate differences in muscle coordination between individuals, the %Total EMG from each muscle was averaged across repetitions. These variables were also used to evaluate participant variation between conditions.

For analysis of shear modulus data, single images were extracted from each elastogram frame (0.7–1.3 Hz) in.PNG format and imported into MATLAB for analysis. Regions of interest (ROI) were optimised for each muscle as described previously (Tier et al. 2021) (Fig. 1A–C). For four participants in ‘Natural’ condition, the deepest region of multifidus was not included in the ROI (see Fig. 1). Average shear modulus was calculated by averaging the Young’s modulus (kPa) across pixels within each ROI, and divided by three (Eby et al. 2013). SWE data were synchronised with EMG using the trigger and data were averaged across 5% force increments (Bin1–Bin6). Change in shear modulus between Bins was calculated as for EMG (ΔBin1−ΔBin6). The minimum value was calculated as the average across frames during rest (force < 1% MVC) and subtracted from all shear modulus values to account for individual variation in baseline muscle shear modulus. If force at rest was not < 1%MVC, baseline values were used from adjacent repetitions. Data for the first frame of each trial were deleted. Additional contractions at 50%MVC with EMG feedback were performed after each task to normalise shear modulus data (shear modulus saturated at 100%MVC). Two participants did not reach 50%MVC EMG amplitude for a muscle. In this case, shear modulus at 50%MVC EMG was extrapolated from regression between EMG and shear modulus between 0 and 30%MVC recorded for another study (Tier et al. 2021).

### Statistical analysis

Statistical analyses were conducted in Stata (version 12, StataCorp LLC, USA). Shapiro–Wilk (skewness/kurtosis) test assessed distribution, and data that were not normally distributed were transformed using log-transformation for EMG data and square root transformation for SWE data.

To evaluate muscle coordination in the ‘Natural’ condition (Aim i) and to compare to trials following feedback (Aim iii), separate generalised linear mixed-effects model (GLM) was used to compare average EMG, change in EMG, and %Total EMG between Muscles (SM, DM or LG), Force Bins (Bin1…Bin6; ΔBin1…ΔBin6) and Conditions (‘Natural’, ‘After SM’, ‘After DM’). Repeated measures were controlled for by modelling subjects as a random effect, hence allowing estimation of independent intercepts. *T* tests with Sidak correction were used for post hoc comparisons.

To evaluate variation in the ‘Natural’ condition within and between participants (Aim ii) and to evaluate changes following feedback (Aim iv), the muscle with the highest EMG at each Force Bin was identified and differences in the %Total EMG between repetitions (within individuals) were compared.

To compare measures of shear modulus and EMG for the two regions of multifidus (Aim v), group and individual data were evaluated. We used GLMs to compare average, change and %Total shear modulus between Muscle (SM, DM), Force Bins (Bin1…Bin6; ΔBin1…ΔBin6) and Condition (‘Natural’ and ‘After SM’), and fitted a regression line to determine whether the %Total shear modulus increased or decreased over Force Bins. Regression analyses were also used to estimate the association between average EMG and shear modulus during ‘Natural’ and ‘After SM’ for each individual. As shear modulus data were only available for SM and DM, %Total EMG was re-calculated excluding LG. The slope of the regression lines was used to compare the EMG vs. shear modulus relationship between muscles, between repetitions of the same condition and between conditions.

## Results

### ‘Natural’ coordination between back muscles during constrained isometric trunk extension (group data)

**Fig. 2:**
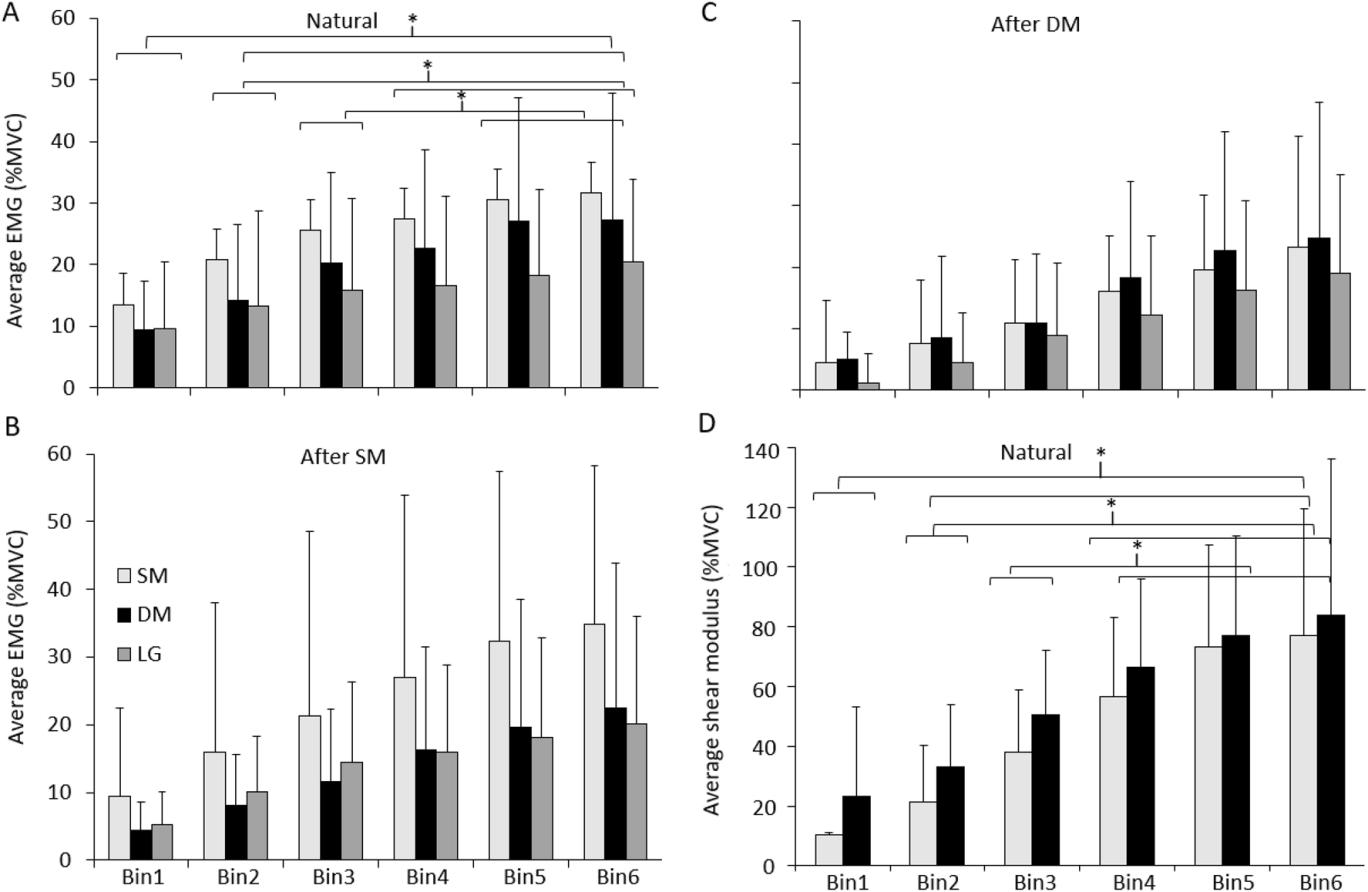
Group average data for average EMG and shear modulus data across Force Bins. Data are shown for average EMG from superficial (SM) and deep (DM) fibres of multifidus and longissimus (LG) during **A** ‘Natural’, **B** ‘After SM’ and **C** ‘After DM’, and for average shear modulus from SM and DM across Force Bins during **D** the ‘Natural’ condition. Mean and SD are shown. *p < 0.05 for comparison between Bins in the ‘Natural’ condition

**Fig. 3:**
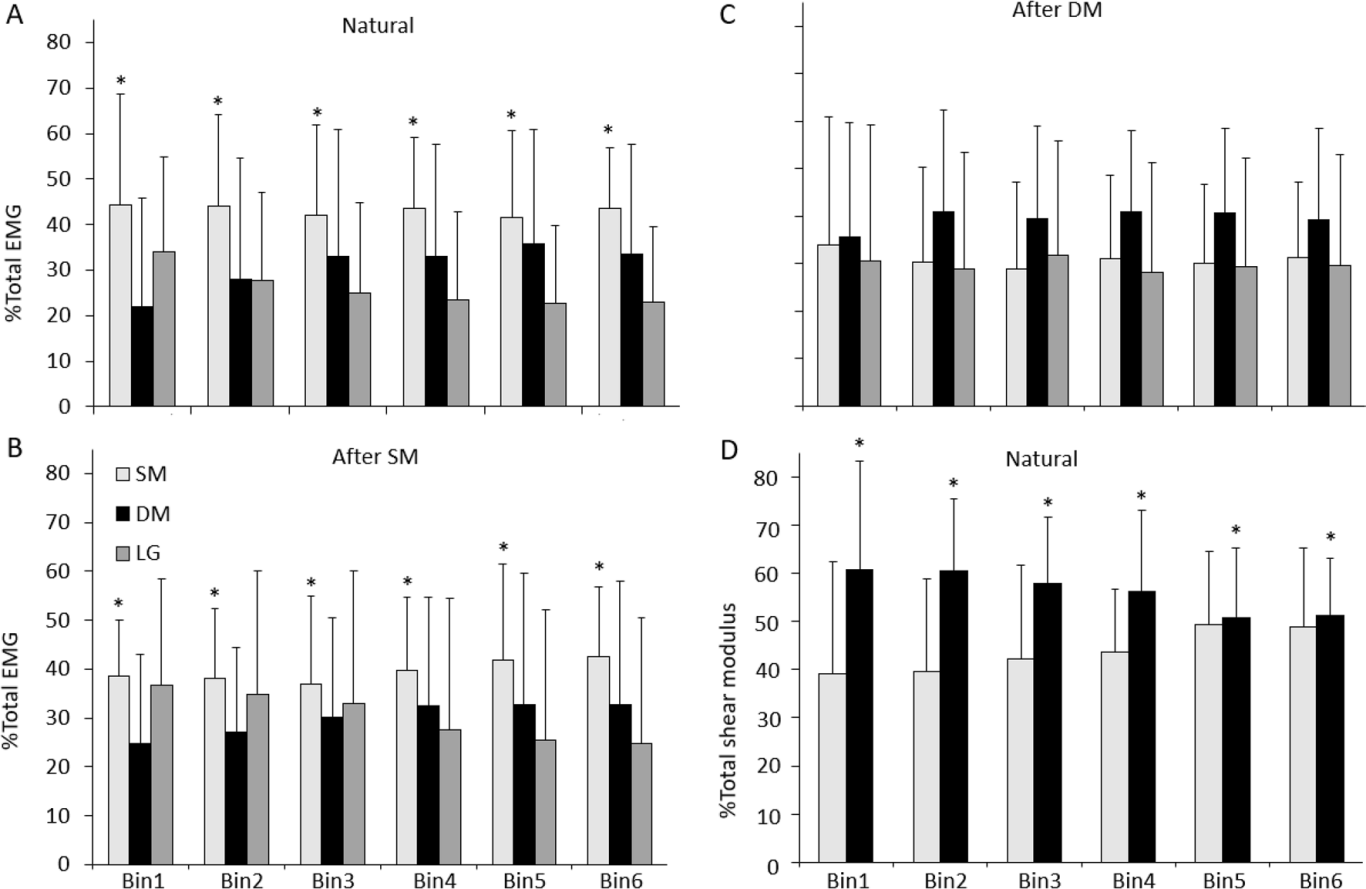
Group average data for %Total EMG and %Total shear modulus across Force Bins. Data are shown for average %Total EMG from superficial (SM), and deep (DM) fibres of multifidus and longissimus (LG) during **A** ‘Natural’, **B** ‘After SM’ and **C** ‘After DM’ and **D** for average %Total shear modulus from SM and DM across Bins during the ‘Natural’ condition. Mean and SD are shown. *p < 0.05 for comparison between muscles in the ‘Natural’ condition for % =Total EMG and % =Total shear modulus and ‘After SM’ condition for %Total EMG

In the ‘Natural’ condition, average EMG increased with force (Main effect of Bin: *p* < 0.001; Fig. 2A) and this increase did not differ between Muscles (Interactions: all *p* > 0.77). Average EMG was greater in Bins2–6 than Bin1, greater in Bins4–6 than Bin2, greater in Bins5–6 than Bin3, and greater in Bin6 than Bin4 (Table 1). Average EMG was significantly greater for SM than DM (Main effect: Muscle—*p* < 0.001, post hoc—*p* = 0.025) and LG (*p* < 0.001), with no difference between DM and LG (*p* = 0.17; Fig. 2A). SM EMG made a significantly greater contribution to %Total EMG than DM (Main effect: Muscle—*p* < 0.001, post hoc—*p* < 0.001) and LG (*p* < 0.001), with no difference between DM and LG (*p* = 0.14) (Fig. 3A). The %Total EMG for each muscle was consistent across Bins (Interaction: Muscle x Bin, *p* = 0.45), suggesting a proportional increase in EMG of each muscle with force, for the group.

**Table 1:** Statistical analysis outcomes

Variable	Muscle	Bin	Condition	Muscle x Bin	Muscle x Condition	Condition x Bin	Muscle x Condition x Bin
Average EMG	< 0.001*	< 0.001*	< 0.001*	0.78	0.003*	0.96	> 0.99
Change EMG	0.021*	0.46	< 0.001*	0.86	0.83	< 0.001*	0.87
%Total EMG	< 0.001*	> 0.99	0.84	0.45	< 0.001*	> 0.99	> 0.99
Average shear modulus	0.018*	< 0.001*		0.90			
Change shear modulus	0.44	0.03*		0.68			
%Total shear modulus	< 0.001*	1.00		0.12			

*p < 0.05

**Fig. 4:**
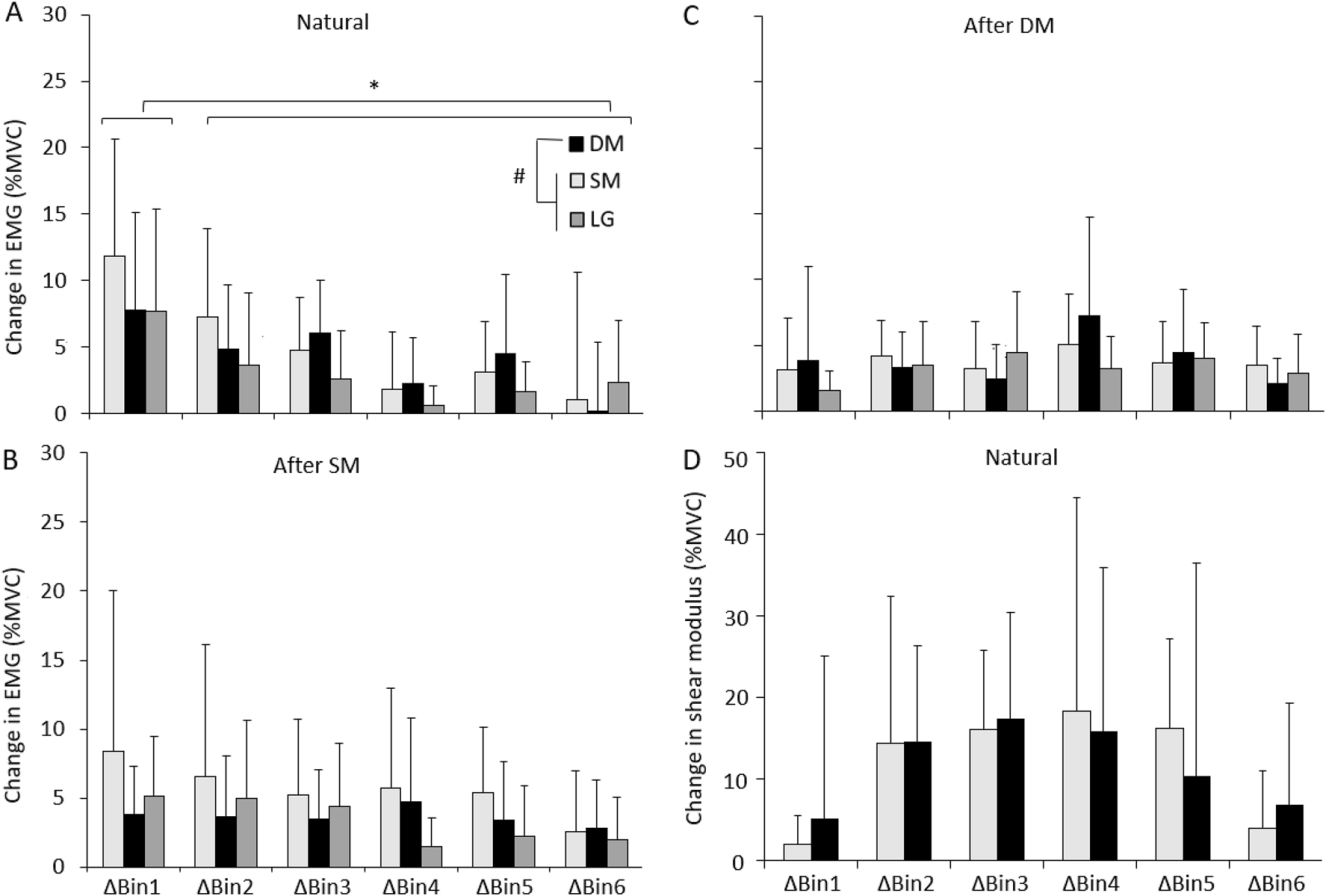
Group average data for change EMG and change shear modulus across Δ =Bins. Data are shown for average change EMG from superficial (SM), and deep (DM) fibres of multifidus and longissimus (LG) during **A** ‘Natural’, **B** ‘After SM’ and **C** ‘After DM’ and **D** for average change shear modulus from SM and DM during ‘Natural’ condition. Mean and SD are shown. *p < 0.05 for comparison between Δ Bins in the ‘Natural’ condition, ^#^*p* < 0.05 for comparison between muscles in the ‘Natural’ condition

Change in EMG between adjacent Bins was greater for SM than LG across all ΔBins (Main effect: Muscle—*p* < 0.021, post hoc—*p* < 0.019; Fig. 4A) but did not differ between SM and DM, or DM and LG (both: *p* > 0.14). Change EMG was greater in ΔBin1 than in other Bins (Interaction: Condition x Bin *p* < 0.001, post hoc *p* < 0.05), with no difference between other ΔBins (all: *p* > 0.32).

### Variation in the ‘Natural’ coordination of back muscles within and between participants

**Fig. 5:**
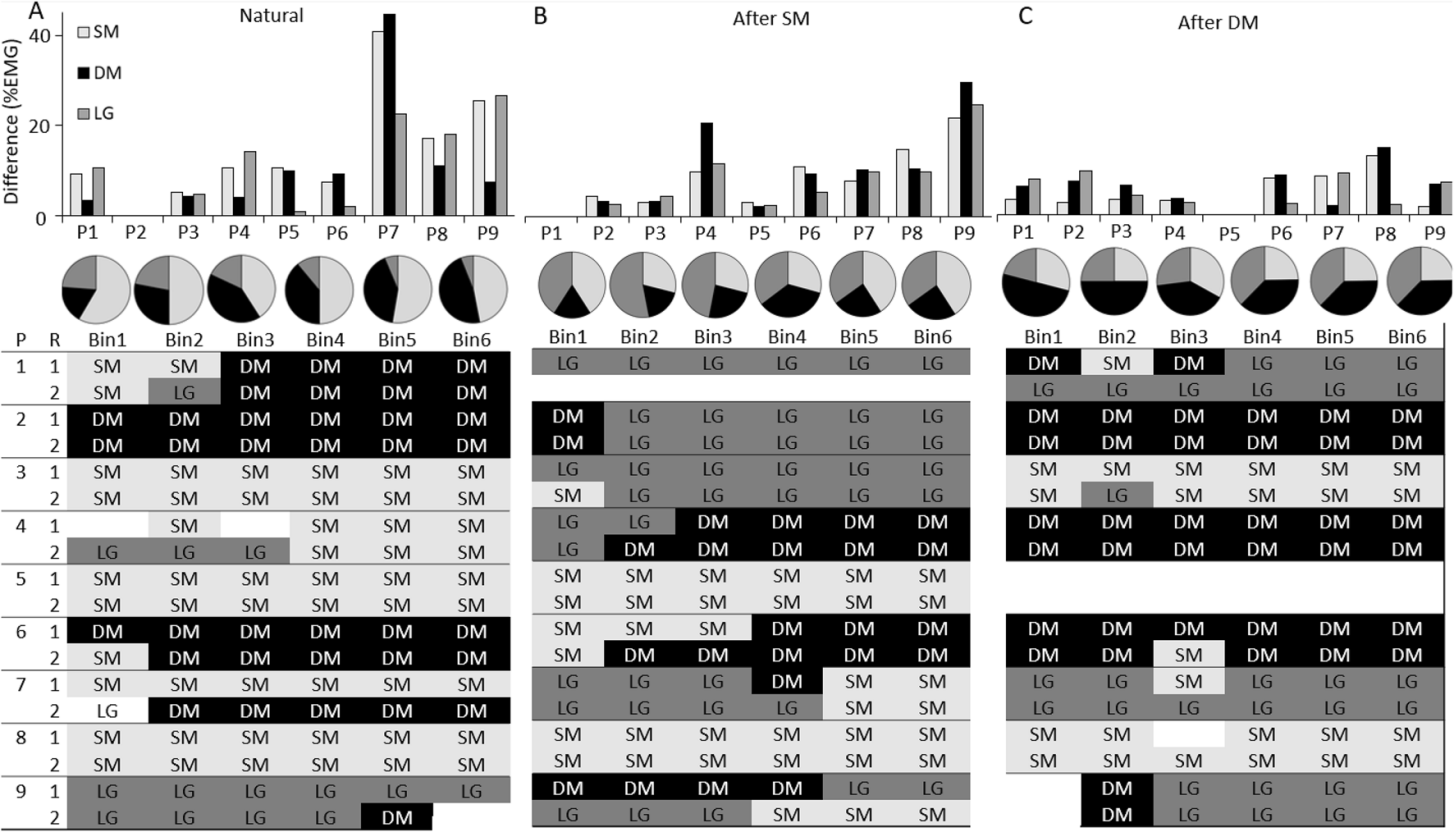
Individual participant data of the absolute between-repetition difference of the %Total electromyography (EMG) from superficial (SM), and deep (DM) fibres of multifidus, and longissimus (LG) during **A** ‘Natural’, **B** ‘After SM’ and **C** ‘After DM’. Pie charts depict the percentage of participants with the muscle with the highest EMG at each Force Bin in each Condition. The table displays the muscle with the highest EMG activity at each Force Bin for each participant for each task repetition at each condition. *P* participant, *R* repetition

Although the average mean difference in %Total EMG between repetitions for each muscle was small (SM: 14%, DM: 10%, LG: 11%; Fig. 5A), this was inconsistent across individuals. For three participants, the difference between repetitions was three times greater than for all the others. This suggests that, although many individuals used a similar coordination strategy between repetitions, for some, the strategy differed substantially.

**Fig. 6:**
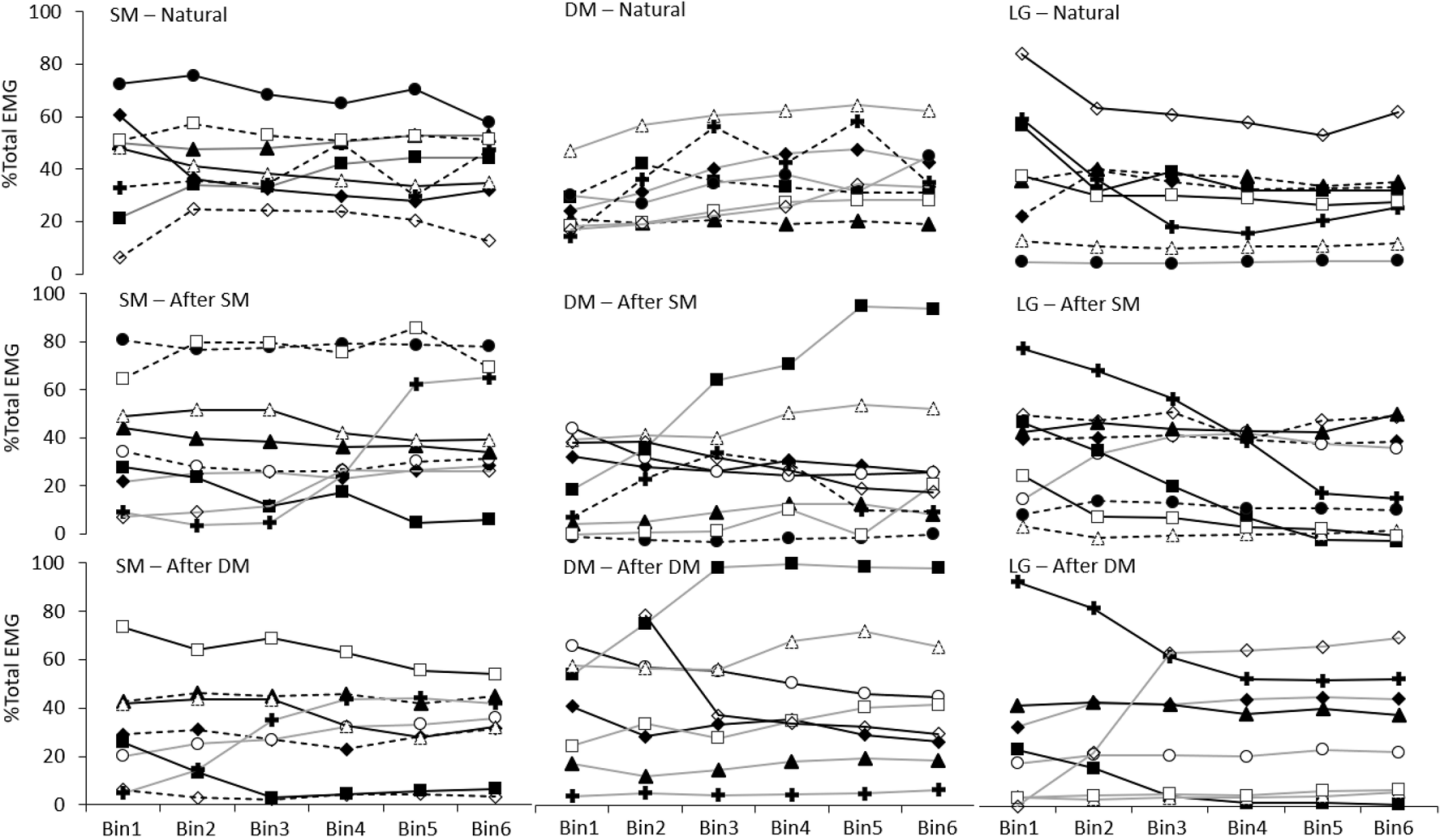
Individual participant data for %Total electromyography (EMG) in each Force Bin. Rows depict data for each condition (‘Natural’, ‘After SM’, ‘After DM’), columns depict data for different muscles—superficial (SM), and deep (DM) fibres of multifidus, and longissimus (LG) and displays the changes in %Total that occurs between muscles in the Conditions

In the ‘Natural’ condition, although group data showed greatest activation of SM and little change in any muscle as %Total EMG with increasing force, data for shown in Fig. 6 demonstrate that, for most individuals, the %Total for each muscle differed between Force Bins with patterns that varied between participants. Across Bins, SM was the muscle with the highest EMG activation for 41–59% participants, (Fig. 5), DM for 18–47% of participants and LG for 6–24%.

### Muscle coordination strategy following EMG feedback of a specific muscle

When the constrained trunk extension task was repeated after practice of the task with visual feedback of SM EMG but no explicit instruction to change strategy, average DM EMG was less than in the ‘Natural’ condition (Interaction: Muscle x Condition, *p* < 0.003, post hoc: *p* < 0.02), but average SM and LG EMG were unchanged (all *p* > 0.39) (Fig. 2A, B). %Total EMG of each muscle was not affected by the feedback (Interaction: Muscle x Condition *p* < 0.001, post hoc: all *p* > 0.33) and, as in the ‘Natural’ condition, %Total SM EMG was greater than other muscles (post hoc: both *p* < 0.02), without a difference between DM and LG (post hoc: *p* = 0.99 Fig. 3B). Compared to the ‘Natural’ condition, the change in EMG between adjacent Bins was lower ‘After SM’ for ΔBin1 (Interaction: Condition x Bin *p* < 0.001, post hoc: *p* < 0.05).

After practice of the task with visual feedback of DM EMG, average EMG of all muscles was less than ‘Natural’ (post hoc: all *p* < 0.02; SM EMG also less than ‘After SM’, *p* < 0.001). Compared with both ‘Natural’ and ‘After SM’, %Total SM EMG decreased ‘After DM’ (Interaction: Muscle x Condition, *p* < 0.003, post hoc: *p* < 0.001), whereas %Total DM EMG increased (post hoc: *p* < 0.01) and %Total LG EMG did not differ between Conditions. %Total DM EMG was greater than LG ‘After DM’ (post hoc: *p* < 0.006).

### Within- and between-participant variation in coordination of back muscles between conditions

#### Within participant

Inspection of data suggests less difference in average EMG between repetitions for trials after EMG feedback than ‘Natural’ (Fig. 5A–C; ‘After SM’—9.9%, 11.7%, and 9.2%, ‘After DM’—6.2%, 7.9%, and 6.5% for SM, DM, and LG, respectively). For some participants, the same muscle had the highest EMG for four or more Bins between repetitions (‘After SM’ *n* = 5; ‘After DM’ *n* = 7), but most participants (*n* = 8) changed the muscle with the highest EMG between conditions.

#### Between participant

Inspection of individual data reveals that muscle coordination varied between participants (Fig. 5). During ‘After SM’, the muscle with the highest EMG was LG for 35–53% of participants, SM for 29–41% and DM for 18–35%. Changes in %Total EMG changes between Bins differed between participants (Fig. 6).

Following DM feedback, DM was the muscle with the greatest %Total EMG, ranging between 38 and 50% (Fig. 3). In contrast to the average group data, visual inspection indicated that %Total EMG increased across Force Bins in four participants for DM, two for SM and five for LG (Fig. 6).

### Comparison between SWE and EMG

#### Comparison of group data—‘Natural’ condition

Similar to average EMG, the average shear modulus (as a proportion of shear modulus at 50% MVC) increased across Bins (Main effect, Bin: *p* < 0.001; Table 1; Fig. 2D), and average shear modulus was greater in Bins2–6 than Bin1, Bins4–6 were greater than Bin2, and Bin6 was greater than Bin3 (post hoc: all *p* < 0.01). In contrast with EMG findings, average normalised DM shear modulus was greater than SM (Main effect: Muscle; *p* < 0.018; Fig. 2D; note that this infers DM shear modulus was a greater proportion of the value at 50% MVC than SM, and not that the absolute shear modulus was greater). DM %Total shear modulus was greater than SM (Main effect: Muscle; *p* < 0.001, Fig. 3D), and did not differ between Bins (Main effect: Bin *p* > 0.99, Interaction: Muscle x Bin *p* = 0.12).

Similar to change EMG, there was a main effect of ΔBins for change shear modulus (*p* = 0.03). However, unlike change EMG, no difference between ΔBins for change shear modulus was detected by post hoc analysis (all *p* > 0.16, Fig. 4). In contrast to EMG, there was no significant difference between muscles in change shear modulus (Main effect: Muscle; *p* > 0.43). Consideration of individual participant data was undertaken to interpret differing observations of EMG and shear modulus.

#### Comparison of individual participant data in the ‘Natural’ condition

**Fig. 7:**
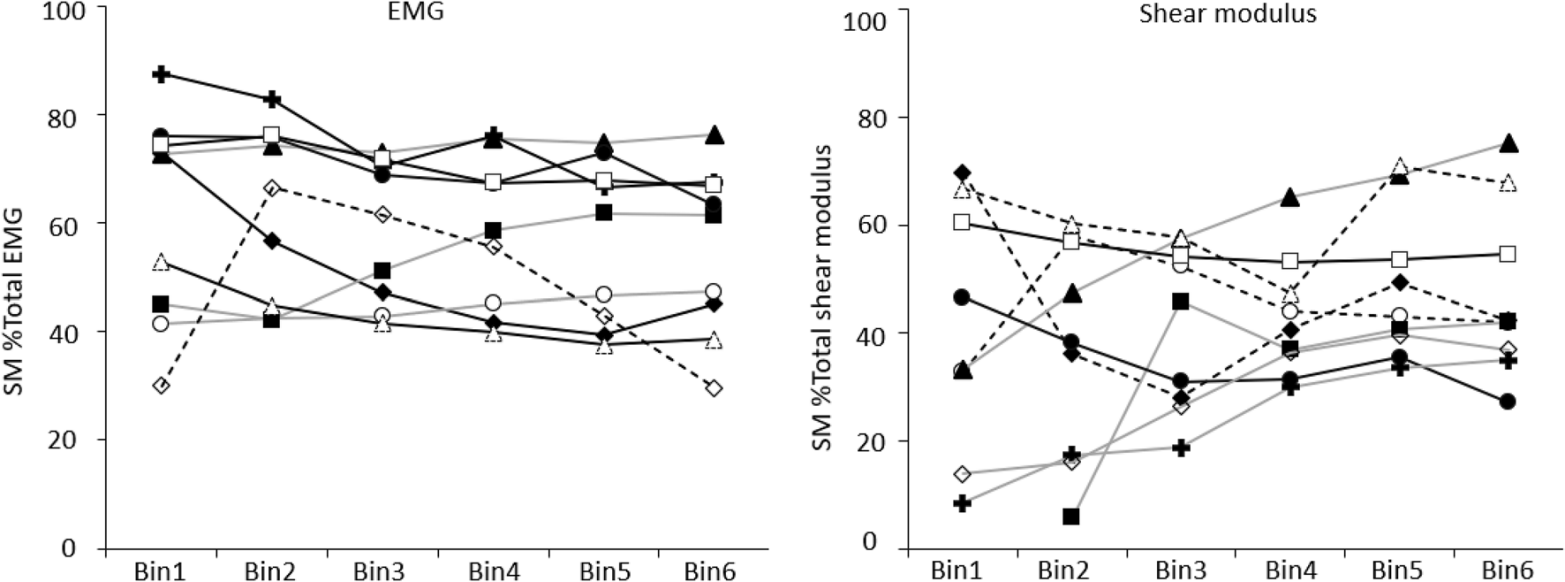
Individual participant data for superficial multifidus (SM) as %Total electromyography (EMG) (left panel) and shear modulus (right panel) during the ‘Natural’ condition across Force Bins (Bin). Shapes depict different participants and are consistent between the two panels

Both EMG and shear modulus showed individual differences in coordination between SM and DM. Although average SM EMG was a greater proportion of its value at MVC than DM in > 50% of participants, average normalised shear modulus SM was a greater proportion of its value at 50% MVC than DM in 33% of participants (Fig. 7). If the relationship between EMG and shear modulus was linear and consistent between muscles, it might be expected that change in EMG or shear modulus would provide similar interpretation for coordination between muscles, but this was not the case. When linear regression was fit to the relationship between EMG and shear modulus data, the relationships were generally positive and linear between 0–30% MVC (66% of participants), but the slopes and intercepts differed substantially between muscles (Table 2).

**Table 2:** Correlation between shear modulus and EMG

Participant	Condition	Repetition	Superficial multifidus Shear modulus vs. EMG	Deep multifidus Shear modulus vs. EMG	Superficial multifidus Between repetition difference	Deep multifidus Between repetition difference
			*r*^2^, slope, intercept	*r*^2^, slope, intercept	Absolute slope difference, % slope difference	Absolute slope difference, % slope difference
1	Natural	1	0.66*, 35.06, 3.05	0.96*, 28.8, 2.68		
		2				
	After SM	1				
		2				
2	Natural	1	0.96*, 17.80, 14.45	0.90*, 12.30, 24.64	1.35, 7%	5.03, 41%
		2	0.94*, 19.16, 18.05	0.45, 7.27, 32.31		
	After SM	1	0.98*, 32.14, 10.98	0.85*, 35.62, 11.35	2.56, 7%	12.10, 33%
		2	0.68*, 29.57, 9.80	0.69*, 23.52, 16.92		
3	Natural	1	0.82*, 29.46, 14.70	0.21, 22.35, 6.55	10.1, 34%	26.42, 118%
		2	0.54, 39.57, 30.32	0.04, − 4.07, 15.66		
	After SM	1	0.39, 17.29, 14.62	0.94*, 12.42, 2.87	8.61, 50%	0.09, 10%
		2	0.64*, 8.68, 12.44	0.78*, 12.5, 2.93		
4	Natural	1	0.98*, 30.3, 3.16	0.89*, 18.48, 2.96	9.16, 30%	14.9, 80%
		2	0.74*, 21.14, 14.90	0.62*, 3.60, 14.28		
	After SM	1	0.32, 0.64, 0.17	0.01, − 0.89, 2.68	9.9, 155%	13.84, 156%
		2	0.66*, 10.55, − 0.43	0.78*, 12.95, − 4.19		
5	Natural	1	0.11, 49.08, 12.19	0.89*, 36.26, 2.86	32.46, 66%	38.80, 107%
		2	0.02, 16.61, 35.29	0.02, − 2.55, 14.33		
	After SM	1	0.74*, 73.77, 54.13	0.80*, 3.25, 4.99	60.4, 80%	0.01, 10%
		2	0.09, 13.37, 67.49	0.61*, 3.23, 2.25		
6	Natural	1	0.31, 22.19, 12.51	0.34, − 68.1, 72.0	2.70, 12%	328.2, 482%
		2	0.10, 24.90, 34.17	0.82*, 260.12, − 27.89		
	After SM	1	0.94*, 27.0, − 0.41	0.73*, 205.52, − 50.23	1.04, 4%	180.7, 88%
		2	0.97*, 28.04, 12.52	0.05, 24.82, 24.39		
7	Natural	1	0.84*, 39.0, 8.84	0.97*, 8.28, − 0.39	33.13, 84%	15.9, 192%
		2	0.99*, 5.87, − 0.11	0.99*, -7.62, 5.44		
	After SM	1	0.91*, 1.00, − 0.04	0.99*, 6.55, − 1.12	0.36, 35%	1.02, 16%
		2	0.77*, 1.363, − 0.28	0.80*, 7.57, − 1.91		
8	Natural	1	0.79*, 32.76, 9.82	0.94*, 28.52, 0.50	7.43, 22%	7.75, 27%
		2	0.84*, 40.19, 9.10	0.90*, 20.77, 4.68		
	After SM	1	0.95*, 24.49, 7.01	0.46, 13.42, − 1.96	1.13, 5%	0.2, 10%
		2	0.93*, 25.62, 3.11	0.73*, 13.22, − 3.16		
9	Natural	1	0.81*, 6.53, 2.68	0.82*, 10.58, 5.80	13.20, 202%	24.35, 230%
		2	0.27, 19.73, 25.78	0.76*, 34.92, − 23.39		
	After SM	1	0.82*, 3.84, 1.55	0.51, 7.90, 12.95	128.30, 334%	2.13, 27%
		2	0.67*, 132.12, − 11.71	0.43, 10.02, 8.58		

*SM* superficial fibres of multifidus

*p < 0.05

**Fig. 8:**
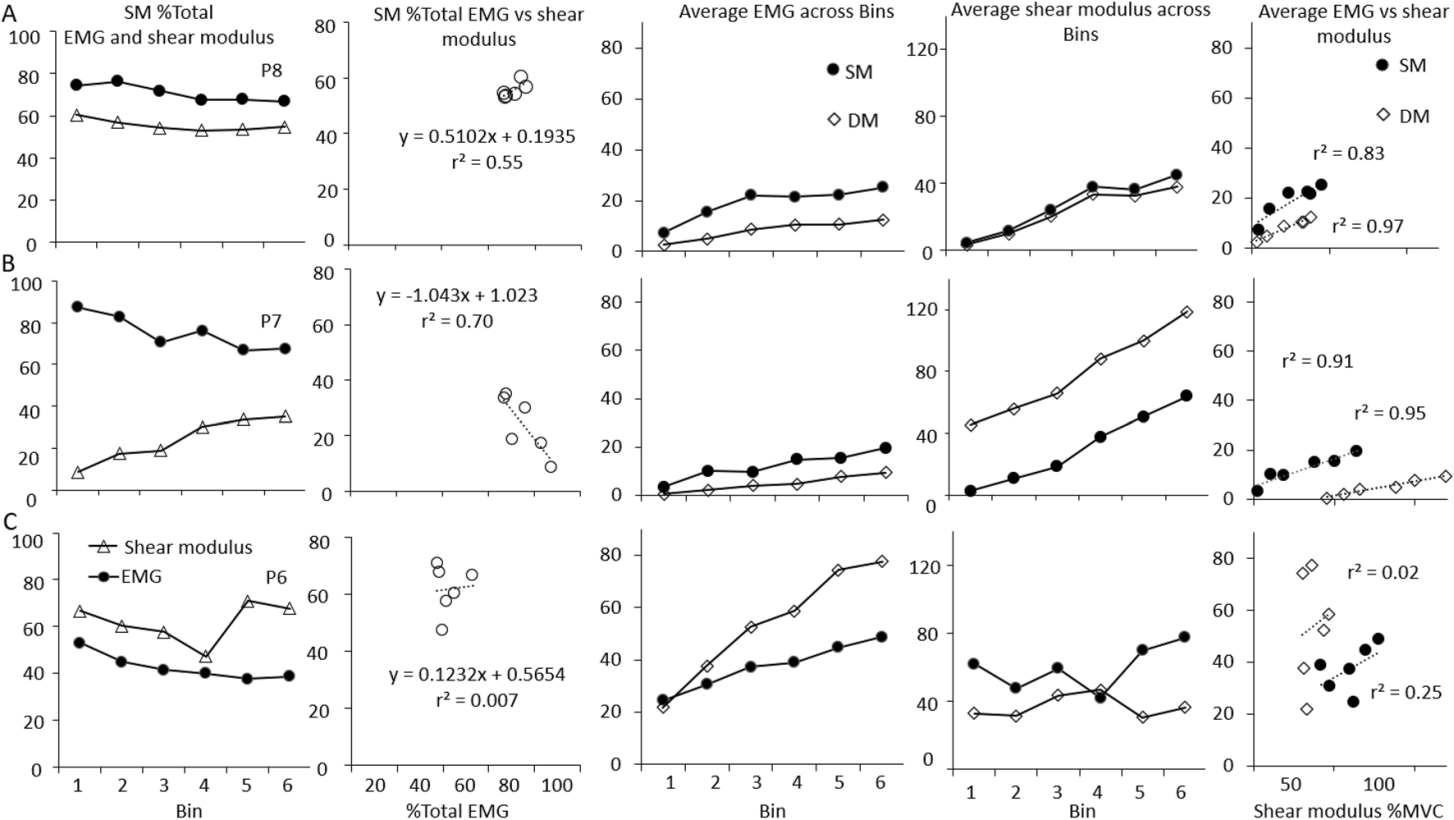
Individual participant data depicting different relationships between electromyography (EMG) and shear modulus. Data are shown for three participants with distinct patterns of muscle activation across rows. **A**–**C** Columns show %Total EMG and %Total shear modulus across Bins for superficial fibres of multifidus (SM); relationship between %Total EMG and %Total shear modulus for SM; average EMG across Bins for SM and deep (DM) fibres of multifidus; average shear modulus across Bins; and the relationship between average EMG and average shear modulus for DM and SM. **A** This participant displays the pattern expressed in the group average data. There is a constant %Total EMG and shear modulus for SM across Bins and a corresponding strong positive correlation between them. Consistent with the uniform SM %Total EMG and shear modulus, both DM and SM average EMG and shear modulus increase with force in a similar manner, and a strong positive correlation between EMG and shear modulus. **B** This participant shows a relationship whereby SM %Total EMG and shear modulus show opposite trends across Bins, and corresponding negative relationship between them. Reflecting the reduction in SM %Total over Bins, the average EMG across Bins for DM increases proportionally more than SM. Average shear modulus shows the opposite trend. The relationship between average EMG and shear modulus for both SM and DM is strong and positive. **C** For SM, this participant shows a small decrease in % Total EMG and a small but inconsistent increase for %Total shear modulus across Bins. Consequently, there is no relationship between these variables. Average EMG across Bins displays DM increasing at a higher rate across Bins than SM and no change in average shear modulus across most Bins with a corresponding absence of correlation between these variables

Data for representative individual participants (Fig. 8) demonstrate different relationships between EMG and shear modulus. Although a positive linear correlation was found for most participants, the slopes and intercepts differed substantially for participants whose EMG and shear modulus show differences in interpretation of coordination of SM and DM muscles. Changes in shear modulus do not always reflect changes in EMG.

#### Comparison of the relationship between EMG and shear modulus between repetitions and conditions

The correlation between shear modulus and EMG was consistently positive, but its slope and intercept varied between participants, muscles, repetitions and conditions (Table 2). The mean (absolute) difference in the slope of the regression line for the group did not differ between conditions (paired *t* tests all *p* > 0.15), but the [mean (SD)] percentage difference in slope varied substantially between participants for repetitions and conditions; SM–100% (122%) and DM– 316% (653%). Within-participant variability between repetitions of the task was also high (range 7–200%; Table 2). Mean within-participant variability (i.e. average difference in slope) for the conditions was [mean (SD)]: ‘Natural’—SM: 13.7 (11.6), DM: 57.7 (102.8) and ‘After SM’—SM: 26.5 (42.8), DM: 26.3 (58.6).

## Discussion

This study investigated the coordination of back muscles during a constrained trunk extension task and the effects of brief exposure to EMG feedback. Although average group data showed that EMG amplitude and shear modulus increased together with force and a consistent relation between muscles across force increments, there was substantial variation between and within individuals. In contrast to the group trend, most participants changed the relative activation between synergist muscles across Force Bins and between repetitions. In addition, coordination between muscles changed after brief exposure to EMG feedback. Although shear modulus data also showed individual differences in coordination strategies, these were often different to those identified by EMG. The relationship between shear modulus and EMG between 0 and 30% MVC was generally linear and positive, but the slopes and intercepts of the regression lines differed between and within participants which has implications for interpretation of the measures.

### Between- and within-individual variation in coordination of back muscles

The spine’s complexity, with many degrees of freedom, implies the availability of many strategies to achieve similar motor goals, according to the concept of redundancy (d'Avella et al. 2003). Selection of a motor solution depends on multiple factors, such as optimisation of metabolic costs (van Dieën 1997), variation in properties of the neuromusculoskeletal system (Tytell et al. 2011) and habitual patterns or motor exploration (Loeb et al. 1999; Ting et al. 2015). Given the constraint of our lumbar spine extension task and anatomy, we expected different individuals to adopt a similar strategy. The task was designed to favour activation of SM, which has long fascicles between L1 and the sacrum and can lordose (extend) the lumbar spine. In contrast, DM crosses as few as two segments and has a smaller moment arm (MacDonald et al. 2006). LG includes components that cross the thoracolumbar junction, and is only activated to low levels during similar extension tasks in sitting (McCook et al. 2009). Although pooled group data showed greater SM EMG, this was not consistent across participants. This variation implies energy efficiency was not the primary factor considered when selecting a motor solution.

Various mechanisms might explain differences in coordination strategies between individuals. For instance, greater activation of DM than SM might imply prioritisation of another aspect of motor function. Although DM is less efficient for lumbar extension due its small moment arm, its activation might be biased to control lumbar segmental stiffness, as suggested by anatomy (MacDonald et al. 2009; Macintosh and Bogduk 1986; Wilke et al. 1995) and mechanics (Ward et al. 2009). It is plausible that the bias towards this goal might differ between individuals, and this is consistent with differences in back muscle coordination *between* individuals in previous studies (Eriksson Crommert et al. 2017).

We also identified unexpectedly high within-participant variation of back muscle coordination. According to the consistent use of a habitual strategy of elbow muscles (de Rugy et al. 2012) and evidence of consistent use of individual coordination strategies of the vastii muscles (i.e. ‘individual movement signatures’) (Avrillon et al. 2021), we anticipated individuals might use different strategies to each other, but retain consistent strategy between repetitions. In contrast, we found high variation in coordination between DM, SM and LG between repetitions even without change to the trunk extension task. It is plausible that the high within*-participant* variability might serve purposeful goals in the trunk such as variation to distribute tissue load (Hodges and Tucker 2011) or to explore new options (Moseley and Hodges 2006). Alternatively, variation might not be purposeful, but instead it might simply be tolerated because its impact on the motor output is low (Ting et al. 2015). The present data cannot distinguish between these alternatives.

### Effect of enhanced (EMG) feedback on back muscle coordination

Brief exposure to EMG feedback before but not during our task and without any instruction to use the feedback to modify their coordination was used to probe the potential of a minor input to induce variation in back muscle coordination during constrained trunk extension. EMG feedback with no instruction to change performance can modify coordination of muscles of the shoulder (Huang et al. 2013) and leg (LeVeau and Rogers 1980). In the present study, most participants (except one) changed the muscle with the greatest activity after exposure to feedback, despite no feedback during the task. This adaptability reinforces the flexibility of strategies in this redundant system (Ting et al. 2015) and the lack of adherence to a simple objective (e.g. efficiency).

### Relationship between shear modulus and EMG for the multifidus muscle

Linear relationships between shear modulus and *force* have been identified for superficial fusiform and bipennate muscles of the arm and hand between 0–80% MVC (Bouillard et al. 2012, 2011) and in deep and superficial elbow flexor muscles (Barron et al. 2022). Linear relationships have also been identified between shear modulus and *EMG* (Nordez and Hug 2010). The present data show linear relationship between shear modulus and EMG for the DM and SM, as we have reported previously (Tier et al. 2021), but here we show substantial variation between and within participants.

A consistent relationship between shear modulus and EMG was reported within individuals for the biceps brachii over a small range (0–15% MVC) (i.e. group mean absolute difference in slope of regression of shear modulus vs. EMG biceps brachii was 0.48) (Nordez and Hug 2010). In contrast, our data show substantial variation in slope and intercept between muscles, participants, repetitions and tasks. Although variation in slope and intercept of the linear correlation between shear modulus and EMG would be expected between muscles and participants, substantial variation between repetitions and tasks was not expected. Support for this observation can be found in the literature. Although not discussed by the authors, inspection of data presented for regression equations by Ates et al. (2015) for abductor digiti minimi (ADM) reveal within-participant variation of similar magnitude (i.e. group mean (SD) absolute difference in slope of regression of shear modulus of ADM vs. little finger abduction torque—12.2 (6.7)) to that identified here (SM shear modulus vs. EMG—13.7 (11.5)).

Several factors could influence the slope and intercept of the linear relationship between shear modulus and EMG for the multifidus muscle. First, continuity of connective tissue within and between muscles creates potential for lateral transmission of force between adjacent muscles (Huijing 1999). It is plausible that between-trial variation of activity in one muscle region may have modified the shear modulus of another region, without any change in EMG in that other region, thus altering the EMG vs. shear modulus relationship. Second, shear modulus of the back muscles is likely to be impacted by compartment pressure (Willard et al. 2012) independent of multifidus EMG, and this might differ between trials secondary to variation in activity of muscles that attach to the thoracolumbar fascia. Third, the complexity of the anatomy of the multifidus, multipennate with multiple muscle fibre directions, close to bone where ‘void’ areas are present and large inter-individual differences in shear modulus recordings at rest (Creze et al. 2017) could present a greater opportunity for error in shear modulus measurement in the multifidus leading to different estimates of the linear relationship.

### Methodological considerations

Several methodological issues require consideration. First, the order of tasks was not randomised, and therefore the impact of fatigue cannot be excluded, although this would have been limited by the rest breaks. Second, there were minor differences in ROIs used for shear modulus between conditions. However, it has been shown that alteration in transducer alignment of < 20° induces little change in shear modulus in the iliotibial band (Besomi et al. 2021), suggesting that minor changes in transducer positioning are unlikely to have impacted our data. Third, when participants could not reach a 50%MVC force contraction, the shear modulus value used for normalisation was estimated from the correlation equation between shear modulus and EMG, which was estimated in a previous study (Tier et al. 2021). Fourth, as SWE maps were sampled at 1 Hz, it is impossible to identify the precise time at which time the measures were obtained with reference to the force and EMG. Fifth, although our restraint of the pelvis and lower limb reduced the potential for leg force to contribute to the task, this was not eliminated. Sixth, as shear modulus will depend on muscle architecture, we cannot conclude whether the absolute muscle stress is greater in one muscle than another. It is important to recognise that our interpretation of SM and DM shear modulus relates to comparison of the value relative that at 50% MVC. Seventh, we did not record EMG from contralateral or antagonist muscles. This might account for some difference between participants, but does not detract from our main conclusion. Although changes in these muscles would likely influence the activity required from the muscles that we measured, the possibility that this varied between repetitions and contexts further strengthens our conclusion that coordination strategy was flexible in this redundant system. Eighth, although the number of participants was small, and we would have had additional power to detect group differences if additional participants were included, our major outcomes arise from the features of within- and between-participant variation from individual participant data which does not depend on a larger sample.

### Implications for further research

The current findings have implications for further research. First, group data poorly reflected coordination strategies of individual participants, suggesting that investigation of inter-muscle coordination requires consideration of individual participant data. Second, coordination of the trunk muscles varies between individuals, and is not predictable based on minimisation of metabolic cost or repetition of habitual patterns. Third, despite the linear relationship between shear modulus and EMG, the relationship was variable, even within an individual. As such, these measures cannot be used interchangeably and future studies should select the most appropriate measurement tool to answer the specific research question (i.e. use of shear modulus to capture passive and active muscle stiffness or EMG for activation).

## Conclusion

This study revealed substantial variation in coordination between back extensor muscles between participants and between repetitions and contexts for individual participants. This highlights highly flexible coordination of back muscles even in a tightly constrained task.

## Data Availability

The datasets generated during and analysed during the current study are available from the corresponding author on reasonable request.

## Abbreviations

BMI: Body mass index
DM: Deep multifidus
EMG: Electromyography
ES: Erector spinae
GLM: Generalised linear mixed-effects model
LG: Longissimus
MVC: Maximum voluntary contraction
RMS: Root mean square
ROI: Region of interest
SD: Standard deviation
SM: Superficial multifidus
SWE: Shear wave elastography
ΔBins: Change bins
